# Effects of Diallyl Trisulfide, an Active Substance from Garlic Essential Oil, on Energy Metabolism in Male Moth *Sitotroga cerealella* (Olivier)

**DOI:** 10.3390/insects11050270

**Published:** 2020-04-29

**Authors:** Meng-Ya Wu, Yi-Yi Ying, Su-Su Zhang, Xue-Gang Li, Wen-Han Yan, Yu-Chen Yao, Sakhawat Shah, Gang Wu, Feng-Lian Yang

**Affiliations:** 1 Hubei Insect Resources Utilization and Sustainable Pest Management Key Laboratory, College of Plant Science and Technology, Huazhong Agricultural University, Wuhan 430070, China; wumengya@webmail.hzau.edu.cn (M.-Y.W.); y15827599531@163.com (Y.-Y.Y.); zhangsusu@webmail.hzau.edu.cn (S.-S.Z.); 17854296832@163.com (W.-H.Y.); yaoyc97@163.com (Y.-C.Y.); Shahentomology@webmail.hzau.edu.cn (S.S.); wugang@mail.hzau.edu.cn (G.W.); 2 Department of Chemistry, College of Science, Huazhong Agricultural University, Wuhan 430070, China; lixuegang@mail.hzau.edu.cn

**Keywords:** diallyl trisulfide, energy substances, metabolism, *Sitotroga cerealella*

## Abstract

This study investigated the effects of diallyl trisulfide (DAT), an active substance from garlic essential oil, on the metabolism of the main energy substances of pre- and postmating males of *Sitotroga cerealella*. Males at 12 h postemergence were fumigated with DAT at a concentration (LC_10_ = 0.010 µL/L) in a glass jar for 7 h. The main energy metabolites from pre- and postmating males were determined, including protein, triglyceride, glycogen, total soluble sugar, trehalose, and trehalase. The contents of total protein and total soluble sugar and the trehalase activity of premating males were significantly increased following DAT treatment, whereas the contents of protein from the accessory gland, triglyceride, glycogen, and trehalose were significantly decreased after treatment. Additionally, after mating, the total protein and soluble sugar contents were significantly increased and the glycogen content was significantly decreased in the treatment group relative to the levels in controls, but there was no significant difference observed in triglyceride, accessory gland proteins, trehalose content, or trehalase activity between the treatment and control groups. Furthermore, the changes in the main energy substances between pre- and postmating in males after the DAT treatment (∆DAT) were smaller than those in the control group (∆CK). This result indicated that DAT can accelerate the rate of metabolism in males at LC_10_, leading to the accumulation of greater levels of total soluble sugar to support life activities and to the increased synthesis of proteins to resist an adverse environment.

## 1. Introduction

The Angoumois grain moth, *Sitotroga cerealella* Olivier (Lepidoptera: Gelechiidae), is a serious stored-grain pest worldwide that endangers the safety of gain storage [1]. The infestation of moth in wheat and rice can reach 56–75%, which reduces the economic and edible value of products. The moth causes damages up to 40% in storehouses in China [2].

To control pest infestation, synthetic pesticides are mainly used. However, the excessive application of these synthetic chemicals leads to environmental problems, risking human health, killing off target organisms, and causing residues and chemical resistance [3,4]. Botanical pesticides have attracted wide attention due to their high efficiency, low residue, and nontoxicity to humans and the environment [5]. Garlic essential oil, as an important type of botanical pesticide, has the effects of contact killing, inducing avoidance, growth inhibition, and toxicity in stored-grain pests [6,7]. Lin et al. [8] found that the main components of garlic essential oil are diallyl trisulfide (DAT, 46.66%), diallyl disulfide (20.31%), and methyl allyl trisulfide (8.95%). Previous studies have shown that garlic essential oil not only affects the life activities of *S. cerealella*, through inhibiting feeding of larvae and reducing female oviposition [2], but also influences insect physiology and biochemistry, by decreasing activity of digestive enzymes [9], accelerating trehalose catabolism, inhibiting protein synthesis, and damaging energy metabolism in *Myzus persicae* (Sulzer) [10].

The energy substances of an insect include carbohydrates, proteins, and lipids. In insects, trehalose accounts for 80–90% of the total sugars in insect haemolymph. It is a protective factor for insects against adversity [11,12]. The insect body is primarily composed of protein. During growth and development, proteins are decomposed into amino acids by proteases, which play critical roles in various metabolic cycles. Fatty acids are stored in fat bodies as triglyceride and play important roles in the cell membrane and energy metabolism. Besides, lipids are the main components of sperm and participate in sperm membrane remodeling, sperm development, and differentiation [13]. 

Insect reproduction is strongly affected by the availability of energy substances [14]. During mating, male insects transfer their accessory glands’ contents to females, which cause changes in female behavior and physiology, and regulate their postmating effects in the female [15]. 

In our laboratory, researchers have demonstrated that DAT treatment at LC_20_ (0.015 µL/L) inhibited the binding of the moth PBP to female pheromones, interfered with the mating behavior of *S. cerealella*, and resulted in the reduction of female oviposition [16]. Furthermore, the pheromone secretion, remating and fertilization rate, and number of eggs produced were all significantly decreased after treatment with DAT at the same dose [17]. However, interestingly, when the dose of DAT was decreased to LC_10_ (0.010 µL/L), DAT continued to impair moth reproduction, such as reducing the quantity and activity of male sperm and inhibiting the synthesis of juvenile hormone and ecdysone (unpublished data). Although there have been many studies of garlic essential oil on insect toxicity and behavior, to our knowledge, few studies reported the effect of garlic essential oil on insect energy metabolism [18]. 

The purpose of this study was to: (1) determine the key energy metabolite, such as protein, triglyceride, glycogen, total soluble sugar, and trehalose, and the trehalase activity of pre- and postmating male moths; (2) explore whether DAT at a dose of LC_10_ (0.010 µL/L) affects the contents of the main energy metabolites in the male moth.

## 2. Materials and Methods

### 2.1. Insect Cultures

The strain of *S. cerealella*, associated with wheat and maintained in a laboratory for the experimental, came from Wuhan, Hubei province, China, where it was collected in 2017. They were raised in a glass bottle 10 cm high and 4 cm in radius. The bottle was packed with 2–3 cm of the substrate, and the insects were maintained in an incubator at 28 ± 1 °C and RH 75 ± 5% with 14: 10 light: dark photoperiod. The pupae were then selected and placed in a glass tube as described by Chang et al., 2020 [17]. The tube was examined daily for newly emerged virgin moths, and male moths were distinguished for subsequent treatment. 

### 2.2. Bioassay

According to the toxic regression equation of garlic active substances against adults *S. cerealella* [19], the value of LC_10_ was calculated and then the mortality was verified. The males were treated with DAT (LC_10_) for 7 h and then placed in the finger tube. Mortalities of DAT were recorded at four days post-treatment. Each treatment had three replicates, and 30 adults per replicate. 

### 2.3. DAT Treatment and Sample Preparation

The experiment was completely designed at random. Males with an emergence time of 12 h were randomly selected and then fumigated with DAT at a dose of 0.010 μL/L (LC_10_) in a 1000 mL glass jar (10 cm in diameter × 13 cm in height), placed in rearing conditions, and kept in dark for 7 h. After fumigation, each male was transferred to a finger tube. The treated males were paired with virgin females that had emerged 4 h earlier and placed in finger-shaped tubes for mating. Virgin adult no-fumigated male moths were used as controls. Two groups of mating experiment were designed: one is untreated male × untreated female; another is DAT-treated male × untreated female. The pre- and postmating samples of total protein, triglyceride, glycogen, total soluble sugar, trehalose, and the activity of trehalase were extracted from the whole body of insects respectively. The pre- and postmating samples of MAGPs (male accessory-gland proteins) were dissected from male accessory glands. All samples were collected in centrifuge tubes precooled with liquid nitrogen for subsequent measurement. 

DAT treatment groups are called DAT groups, which include treated males and treated males after mating with untreated females. Blank treatment groups are called CK groups, which include untreated males and untreated males after mating with untreated females.

### 2.4. Determination of Total Protein and MAGPs Contents

A BCA protein concentration determination kit (Biosharp Technology Co., Beijing, China) was used to determine protein content. A standard curve was produced with bovine-serum protein. Five biological replicates (10 insects per replicate) were established and the absorption at 562 nm was measured four times for each replicate.

The method of determination of MAGPs content is the same as that of measurement of total protein content. Three biological replicates were established, with 100 male accessory glands per replicate and the absorption at 562 nm was measured four times for each replicate.

### 2.5. Determination of Triglyceride (TG) Content

A TG assay kit (Nanjing Jiancheng Bioengineering Institute, Nanjing, China) was used to determine triglyceride content. A standard curve was produced with TG standard solution. Three biological replicates were established, with 50 insects per replicate and the absorption at 510 nm was determined four times for each replicate.

### 2.6. Determination of Glycogen Content

A glycogen assay kit (Comin Biotechnology Co., Ltd. Suzhou, China) was used to determine glycogen content. A standard curve was produced with a standard glucose solution. Five biological replicates were established, with 10 insects per replicate and the absorption at 620 nm was measured four times for each replicate.

### 2.7. Determination of Total Soluble Sugar Content

A standard curve was produced as follows: 0, 200, 400, 600, 800, or 1000 µL of 100 µg/mL glucose standard solution was added to a 2.0 mL centrifuge tube and mixed thoroughly. If less than 1000 µL was added, phosphate buffer solution (PBS) was added to a final volume of 1000 µL. The solution was centrifuged. Next, 1000 µL of 10% trichloroacetic acid solution was added to each centrifuge tube, and the tube was maintained at room temperature for 5 min. The solution was then centrifuged at 2500 rpm for 5 min. A 5 mL glass tube was precooled, 200 µL of supernatant was added, and 800 µL of 0.2% sulfuric acid–anthrone reagent was added. The mixture was lightly shaken five times. The tube was then immersed in a boiling water bath for 10 min, followed by cooling in ice water for 20 min. The absorbance at 620 nm of 200 µL of the solution in a 96-well plate was determined and the standard curve was constructed. 

Sample preparation and determination were conducted as follows: each sample was centrifuged at 2500 rpm for 10 min, and all of the supernatant was collected in a 1.5 mL centrifuge tube. Next, 10% trichloroacetic acid solution of the same volume as the supernatant was added, and the tube was centrifuged at 5000 rpm for 5 min. The determination steps were the same as described above. Five biological replicates were established, with 10 insects per replicate, and the absorbance at 620 nm was repeated four times for each replicate.

### 2.8. Determination of Trehalose Content

A trehalose assay kit (Comin Biotechnology Co., Ltd. Suzhou, China) was used to determine trehalose content. A standard curve was made with standard trehalose solution. Five biological replicates were established, with 10 insects per replicate, and the absorption at 620 nm was determined four times for each replicate.

### 2.9. Determination of Trehalase Activity

A trehalose assay kit (Comin Biotechnology Co., Ltd. Suzhou, China) was used to determine trehalase activity. Five biological replicates were established, with 50 insects per replicate, and the absorption at 505 nm was measured four times for each replicate.

### 2.10. Statistical Analysis

The data on contents of protein, triglyceride, glycogen, total soluble sugar, and trehalose, trehalase activity, and the energy substances between pre- and postmating in CK males (∆CK) and DAT males (∆DAT) were calculated respectively. Independent-sample Tukey’s tests were performed for all data comparing the mean of the treatment and control group (*p* < 0.05) using SPSS v19.0 (SPSS Inc., Chicago, IL, USA). All the figures were produced using GraphPad Prism 5 (GraphPad Software Inc., San Diego, CA, USA). The data were presented as the mean ± standard error of mean (mean ± SEM). The differences were considered statistically significant if *p* < 0.05. ∆CK denotes the difference between “postmating” and “premating” values; ∆DAT denotes the difference between “postmating” and “premating” values.

## 3. Results

### 3.1. Bioassay

According to the toxic regression equation of garlic active substances against adults *S. cerealella*, the value of LC_10_ was calculated as 0.010 µL/L. The mortalities of the CK and DAT groups were 6.67% and 16.67%, respectively. After adjustment, the mortality of diallyl trisulfide at the concentration of 0.010 μL/L against male moths was 10.71%.

### 3.2. Effect of DAT on Protein Content in Pre- and Postmating Males

**Figure 1:**
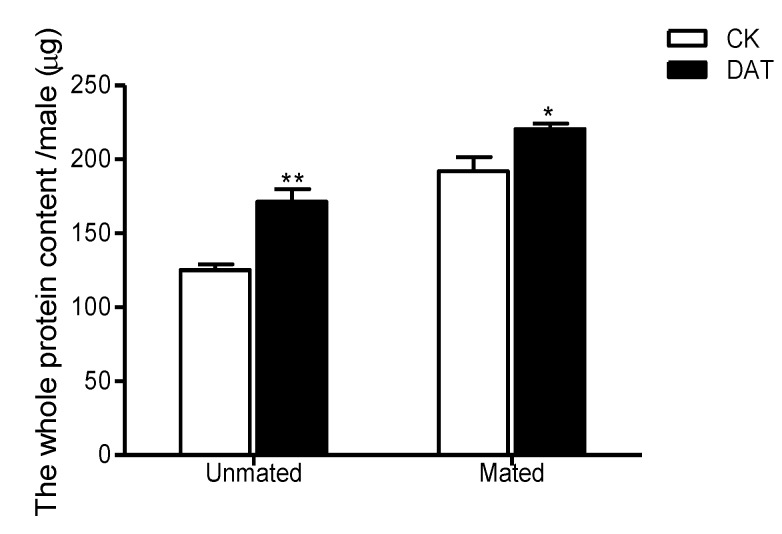
The whole protein content of males was determined in the control (CK) group (at 19 h after emergence) and diallyl trisulfide (DAT) group (treated with 0.01% diallyl trisulfide for 7 h at 12 h after emergence) both before and after mating. The data in the figure are presented as the mean ± SEM. Asterisks depict significant differences relative to the control treatment (*, *p* < 0.05; **, *p* < 0.01).

**Figure 2:**
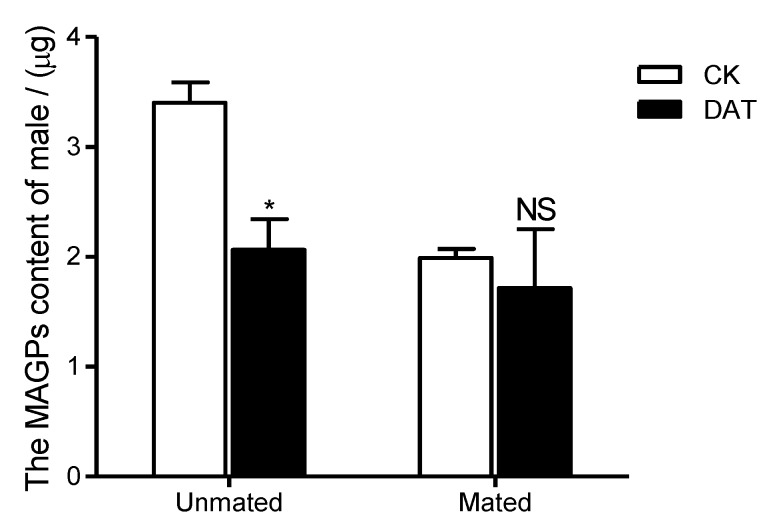
The male accessory gland protein (MAGP) content of males was determined in the CK group (at 19 h after emergence) and DAT group (males treated with 0.01% diallyl trisulfide for 7 h at 12 h after emergence) both before and after mating. The data in the figure are presented as the mean ± SEM. Asterisks depict significant differences relative to the control treatment (NS, no significant difference, *, *p* < 0.05).

The standard curve of protein was y = 0.0002x + 0.0674 (where x is protein concentration (µg/mL), and y is measured absorbance; R^2^ = 0.9967). Using the standard curve, the protein contents of males before and after mating were calculated. The results showed total protein content was significantly increased in premating males treated with DAT, reaching 171.34 ± 8.62 µg/insect relative to that of the premating control males, 125.10 ± 4.00 µg/insect (*F* = 4.932, *df* = 8, *p* = 0.001). A similar pattern was observed for postmating protein content, with a significant difference observed between the treated and control males (*F* = 3.653, *df* = 8, *p* = 0.023). The total protein content of treated postmating males was 220.52 ± 3.68 µg/insect, whereas that of control males was 192.05 ± 9.49 µg/insect (Figure 1). The total protein content of postmating males was significantly higher than that of the premating males in both the CK group (*F* = 3.0801, *df* = 8, *p* < 0.01) and the DAT group (*F* = 5.809, *df* = 5.413, *p* = 0.003). However, different patterns were observed for the MAGPs (Figure 2). After fumigation, the content of MAGPs in premating males was 2.08 µg/insect, which was significantly lower than that in untreated males (3.40 µg/insect, *F* = 4.662, *df* = 4, *p* = 0.01). After mating, MAGP content in both the control and treated groups was decreased relative to that before mating, although the difference between the two groups after mating was not significant (CK = 1.99 µg/insect and DAT = 1.71 µg/insect, *F* = 7.854, *df* = 2.033, *p* = 0.661). The MAGP content of postmating males was significantly higher than that of premating males in the CK group (*F* = 2.554, *df* = 4, *p* < 0.001), but there was no difference in MAGP content between premating and postmating males in the DAT group (*F* = 1.758, *df* = 4, *p* = 0.594).

### 3.3. Effect of DAT on the Triglyceride Content of Pre- and Postmating Males

**Figure 3:**
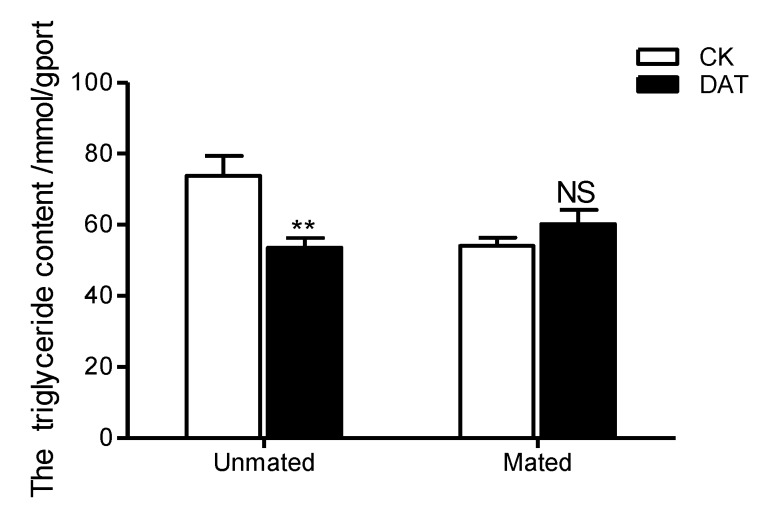
The triglyceride content of males was determined in the CK group (at 19 h after emergence) and DAT group (males treated with 0.01% diallyl trisulfide for 7 h at 12 h after emergence) both before and after mating. The data in the figure are presented as the mean ± SEM. Asterisks depict significant differences relative to the control treatment (NS, no significant difference, **, *p* < 0.01).

After DAT fumigation, the triglyceride content of males before mating was 53.52 mmol/g (Figure 3), which was significantly lower than that of untreated males before mating (CK = 73.76 mmol/g, *F* = 1.032, *df* = 10, *p* = 0.004 mmol/g). However, after mating, there was no significant difference between the DAT (60.20 mmol/g) and CK groups (54.05 mmol/g; *F* = 0.826, *df* = 6, *p* = 0.313). We also found that the triglyceride content of the control males was significantly lower after mating than before mating (*F* = 3.896, *df* = 5, *p* = 0.036), whereas this pattern was not observed in the treatment group (*F* = 0.01, *df* = 11, *p* = 0.184).

### 3.4. Effect of DAT on the Glycogen Content of Pre- and Postmating Males

**Figure 4:**
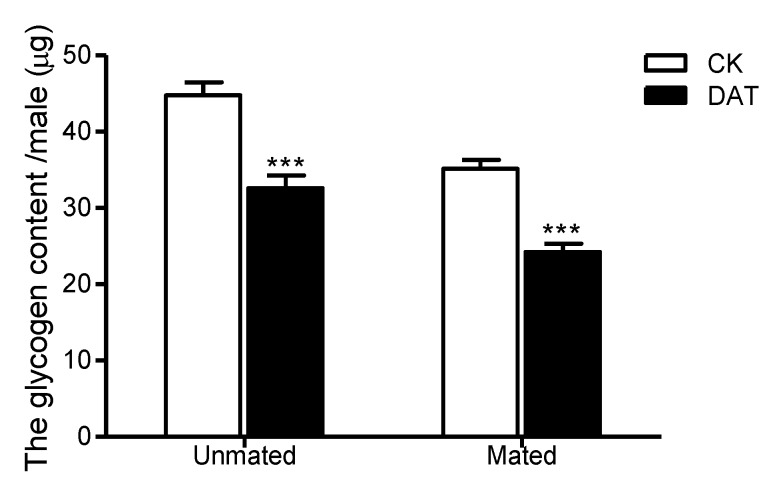
The glycogen content of males was determined in the CK group (at 19 h after emergence) and DAT group (males treated with 0.01% diallyl trisulfide for 7 h at 12 h after emergence) both before and after mating. The data in the figure are presented as the mean ± SEM. Asterisks depict significant differences relative to the control treatment (***, *p* < 0.001).

The standard curve of glycogen was y = 0.0054x + 0.1119, where x is the concentration of the glucose standard (µg/mL), y is the absorbance at 620 nm; the correlation coefficient R^2^ was 0.9979. After fumigation, the glycogen content of the whole body of pre- or postmating males could be obtained from the standard curve. The glycogen content of the whole body of premating males was decreased to 32.60 ± 1.68 µg/insect (Figure 4), which was significantly lower than that of the control (44.79 ± 1.68 µg/insect, *F* = 2.477, *df* = 18, *p* < 0.001). After mating, in both the control and treated groups, the glycogen content was significantly decreased postmating relative to that premating. In the CK group, the content was decreased to 35.13 ± 1.19 µg/insect (*F* = 0.310, *df* = 18, *p* < 0.001), and in the DAT group, it was decreased to 24.22 ± 1.09 µg/insect (*F* = 9.986, *df* = 18.648, *p* = 0.001); the difference between the two groups postmating was significantly different (*F* = 0.001, *df* = 21, *p* < 0.001).

### 3.5. Effect of DAT on the Total Soluble Sugar Content of Pre- and Postmating Males

**Figure 5:**
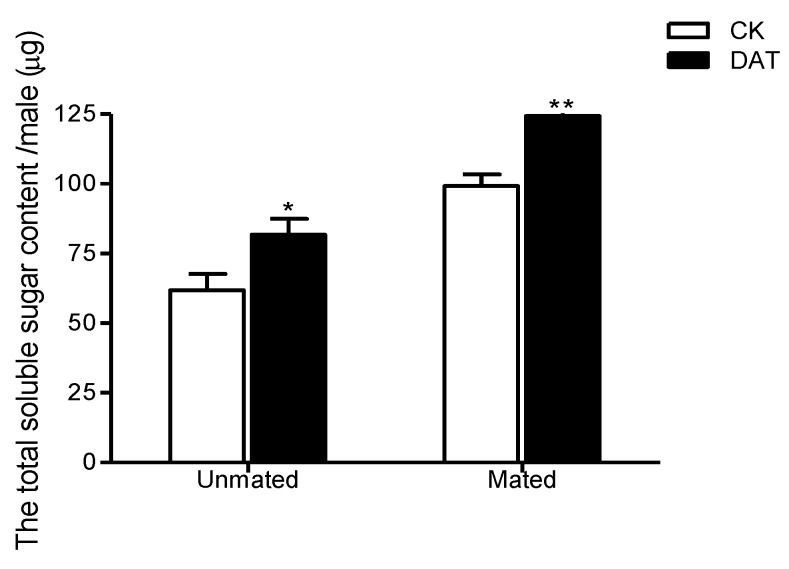
The total soluble-sugar content of males was determined in the CK group (at 19 h after emergence) and DAT group (males treated with 0.01% diallyl trisulfide for 7 h at 12 h after emergence) both before and after mating. The data in the figure are presented as the mean ± SEM. Asterisks depict significant differences relative to the control treatment (*, *p* < 0.05; **, *p* < 0.01).

The total soluble-sugar content of the pre- and postmating males was determined from the standard curve of y = 0.0027x + 0.1155 (where x is the concentration of the glucose standard (µg/mL), and y is the absorbance at 628 nm; R^2^ = 0.9958). As shown in Figure 5, the total soluble sugar content of premating males was 61.84 ± 5.78 µg/insect in the control group and 81.68 ± 5.74 µg/insect in the DAT group; this difference was significant (*F* = 0.03, *df* = 8, *p* = 0.041). After mating, the total soluble sugar content was significantly increased relative to that premating in both the control males, reaching 99.20 ± 4.20 µg/insect (*F* = 0.613, *df* = 8, *p* = 0.001), and in the treated males, reaching 124.36 ± 4.85 µg/insect (*F* = 0.02, *df* = 8, *p* < 0.001). The difference between the two groups postmating was significant (*F* = 0.223, *df* = 8, *p* = 0.004).

### 3.6. Effect of DAT on the Trehalose Content of Pre- and Postmating Males

**Figure 6:**
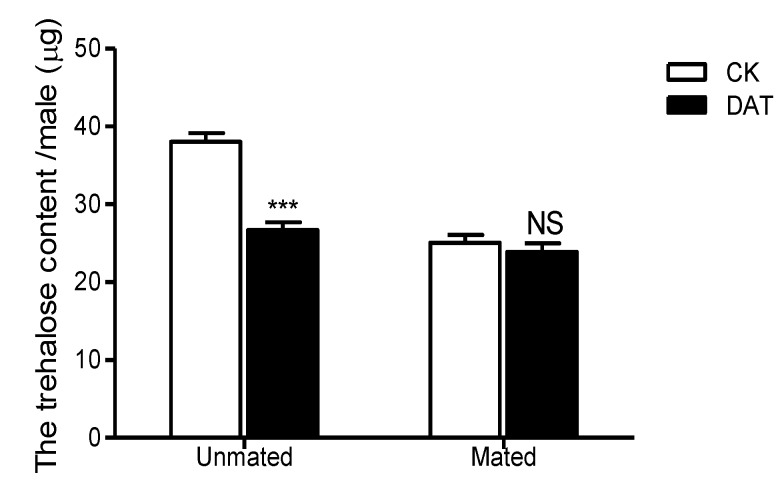
The trehalose content of males was determined in the CK group (at 19 h after emergence) and DAT group (males treated with 0.01% diallyl trisulfide for 7 h at 12 h after emergence) both before and after mating. The data in the figure are presented as the mean ± SEM. Asterisks depict significant differences relative to the control treatment (NS, no significant difference, ***, *p* < 0.001).

The trehalose contents of the pre- and postmating males, shown in Figure 6, were determined from the standard curve (y = 0.0045x + 0.0729, where x is the concentration of trehalose standard (µg/mL), and y is the absorbance at 620 nm; R^2^ = 0.9968). The trehalose content of premating males treated with DAT was 26.70 ± 0.97 µg/insect, which was significantly lower than that of the premating control males (38.00 ± 1.14 µg/insect, *F* = 0.908, *df* = 19, *p* < 0.001). After mating, trehalose content was decreased relative to that premating in both the control and treated groups. It decreased to 25.07 ± 0.99 µg/insect in the CK group, representing a significant difference from that premating (*F* = 0.237, *df* = 19, *p* < 0.001); in the DAT group, it decreased to 23.88 ± 1.10 µg/insect, although the difference from the premating value was not significant (*F* = 0.603, *df* = 23, *p* = 0.068). Additionally, there was no significant difference in trehalose content between the treated and control groups after mating (*F* = 0.373, *df* = 24, *p* = 0.433).

### 3.7. Effect of DAT on the Trehalase Activity of Pre- and Postmating Males

**Figure 7:**
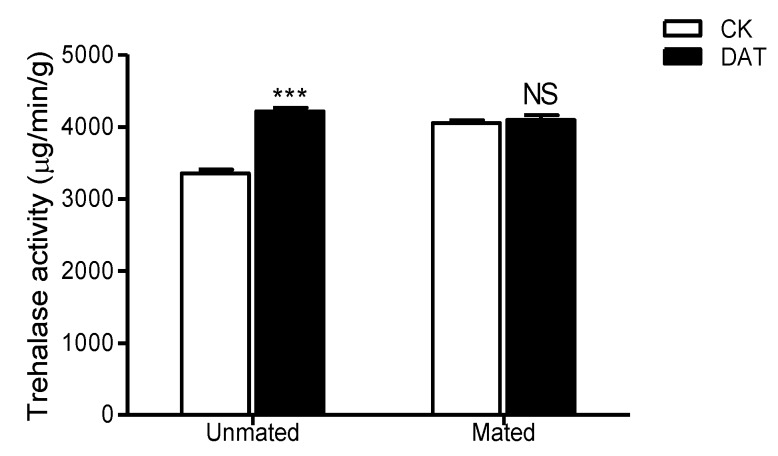
The trehalase activity of males was determined in the CK group (at 19 h after emergence) and DAT group (males treated with 0.01% diallyl trisulfide for 7 h at 12 h after emergence) both before and after mating. The data in the figure are presented as the mean ± SEM. Asterisks depict significant differences relative to the control treatment (NS, no significant difference, ***, *p* < 0.00).

The activity of trehalase was 3352.5 ± 51.81 µg/min/g before fumigation (Figure 7) and increased to 4215.57 ± 48.07 µg/min/g after DAT fumigation, representing a significant difference (*F* = 0.005, *df* = 16, *p* < 0.001). After mating, the trehalase activity of the control males had increased to 4051.19 ± 40.41 µg/min/g (*F* = 1.406, *df* = 16, *p* < 0.001), whereas that of the treated males had decreased to 4092.57 ± 69.56 µg/min/g (*F* = 2.390, *df* = 18, *p* = 0.163), the difference between the two groups after mating was not significant (*F* = 5.823, *df* = 14.454, *p* = 0.615). Furthermore, we found no significant difference in trehalase activity between the premating and postmating males in either the control or treated group. 

Based on the above results, we can conclude that the differences in some of the major energy substances between pre- and postmating males treated with DAT were smaller than the corresponding differences in the controls. Some of these differences were significantly different, i.e., those in the contents of total protein, MAGPs, triglyceride, and trehalose, and in trehalase activity. However, no significant differences were observed in the glycogen and total soluble sugar contents (Table 1). 

## 4. Discussion

Metabolites such as sugar, lipid, and protein are necessary substances for energy in all the life stages of insects and are fundamental to many physiological processes in insects, such as reproduction, flight, molting, and defense against pathogens [20,21]. 

Studies have shown that insecticides stimulate insect metabolism. Sublethal doses of cypermethrin affected the total body weight and significantly declined the glycogen and lipid contents of *Pimpla turionellae* (L.) while they increased the protein of *Spodoptera litura* Fabricius [22,23]. However the effects of sublethal doses of insecticides on insects vary with insect species and insecticide types. In the same way, the effects of insecticides on the metabolism of major energy substances in insects are also different [24,25]. Previous studies have shown that the effect of different sublethal concentrations (LC_10_, LC_20_, LC_30_, LC_40_) of spinosad and the impact petroleum ether extract of *Artemisia annua* Linn. and *Azadirachta indica* A.Juss. on the content of protein, lipid, carbohydrate, glycogen of *Glyphodes pyloalis* Walker, *Anopheles stephensi* Liston, and *Culex quinquefasciatus* larvae were different respectively [26,27].

Proteins are important energy substances used to support insect life activities and affect their fitness-associated characteristics such as fecundity, growth rate, and body size [28]. The result of the present study showed that the protein contents of males significantly increased following treatment with DAT, whereas the accessory-gland protein contents significantly decreased. However, what types of proteins were changed in structure or content is unknown. Insecticides have strong influences on protein synthesis in insects. Proteins such as heat-shock proteins, ecdysone receptor, and vitellogenin are essential for insect growth, development and reproduction [29,30]. When an insect encounters an adverse environment, it synthesizes a large number of stress-resistance proteins. For example, heat-shock protein, a type of stress protein, is induced by high or low temperature, high salt, nutrient starvation, pesticides, and other stimulations. Insecticides can induce the overexpression of heat shock protein family genes in insects to enhance adversity resistance [31,32,33]. Under the adverse condition of fumigation with DAT, a large number of proteins are synthesized by the moth to resist adversity, and this may be one of the reasons for the increase in protein content of the moth. However, what types of proteins were synthesized by the moth to resist the adversity should be further studied in future. 

MAGPs are the main component of accessory-gland contents, affecting the reproductive capacity of insects. They also represent the majority of the seminal-fluid proteins [34]. Gillott [35] has placed accessory-gland secretions into three groups: small peptides, molecules of 200–400 amino acids that are commonly glycosylated, and large proteins. In insects, MAG secretions are known to improve males’ reproduction by a variety of mechanisms, such as sperm mobility, sperm storage, and stimulation of oviposition [35]. MAGPs are closely related to insects’ reproductive capacity. Triazophos treatment has been shown to significantly increase the amount of accessory-gland proteins in *Nilaparvata lugens* (stal) and the transfer of accessory-gland contents to females during mating, which stimulate female reproduction [15]. In the present study, the decrease of protein contents in MAGs was induced by DAT, which further indicated that DAT have no resistance to the moth at present. Also the decrease in treated males (∆DAT) was lower than that of control males (∆CK), indicating that the males treated with DAT did not transfer substantial amounts of MAGPs during mating. A previous study by Ge et al. [36] showed that the mechanism of insecticide-induced increase in MAGP content in *N. lugens* may be associated with the accumulation of more energy in insects developed from nymphs fed on rice plants treated with triazophos and deltamethrin. In our study, because of the nonfeeding characteristics of moth adults, we think that DAT could accelerate energy metabolism and synthesize a large number of proteins to resist adversity without any resistance to the moth, so there were not enough substances to synthesize MAGPs for reproduction. However, in our study, we detected only total proteins and MAGPs, so further research is needed to explore whether DAT affects the synthesis of these reproduction-related proteins.

Along with proteins, trehalose plays a vital role in responding to adversity. Trehalase not only is an important enzyme involved in sugar metabolism but also exists in the reproductive structures of male insects, such as the bean-shaped accessory glands (BAGs), testes, seminal vesicles, vas deferens, tubular accessory glands (TAGs), and ejaculatory ducts [37]. We found that trehalase activity was significantly increased after DAT fumigation, which accelerated trehalose catabolism.

Furthermore, the utilization rates of trehalose and glycogen were significantly increased, and the stored sugar was converted into glucose, resulting in a significant increase in total soluble-sugar content to maintain life activities under the adverse environment and to support active protein synthesis. Besides, the trehalase protein from the reproductive glands may be delivered to the spermatophore. Trehalase activity in the fat body and haemolymph of silkworm was observed to significantly increase following treatment with fenitrothion and ethiophos [38]. In insects, the seminal sugars are mixtures. The spermatophore of *Tenebrio molitor* contains glucose and trehalose [34]. What roles do trehalose and trehalase play in the reproductive system? Whether they function in sperm activation as they are transferred from the male reproductive glands to the female spermatophore during mating remains to be explored. 

In addition to trehalose, triglyceride and proline are among the most common metabolic raw materials of insects. Fatty acids are stored as triglyceride within the lipid droplets of the fat body. Triglyceride play important roles in cell formation and energy metabolism, including the provision of metabolic fuel to flight muscles, the provision of lipids to the ovaries, sex-hormone synthesis and spermatozoon development [13,39,40]. Our study showed that triglyceride content significantly decreased after treatment with DAT. This decrease may have been due to the acceleration of lipid metabolism in males treated with DAT. Similarly, Lohar et al. reported the reduction in lipid contents when *Tenebrio molitor* was exposed to malathion [41]. Furthermore, when *Anopheles stephensi* Liston larvae were treated with plant extracts the total lipid was reduced, which suggested that was due to physiological-stress conditions induced by the extracts [42]. The decrease in the lipid quantity may be due to shift in energy metabolism towards lipid catabolism as a result of insecticidal stress induced by DAT. Triglyceride, as the main energy substance, are decomposed into glycerol and fatty acids, releasing ATP and other metabolic intermediates in the metabolic process. These metabolites are used as raw materials for the synthesis of other substances. The metabolism of energy substances is important for insect life. The three types of energy substances are interrelated and transformed. 

## 5. Conclusions

In conclusion, our research showed that DAT treatment could disorder energy metabolism, for example, the increase in certain biochemical components by DAT suggests metabolic-activity disturbance, while the decrease in different components shows that DAT may affect the digestion enzymes and a certain behavior of the moth. DAT also affects physiological and biochemical factors, such as the activity of sperm and hormone synthesis, ultimately causing a decrease in female oviposition. Further studies are needed to identify the dynamic changes of the main energy substances of males at different times after fumigation and to determine whether these changes affect sperm quantity, activity, and hormone titers. Garlic essential oil is an important kind of botanical pesticide, the green and environmental characteristics of which meet the goals of sustainable development; it has great development potential and broad application prospects [43,44]. Therefore, it is of great significance to study the mechanisms underlying its inhibition of the reproductive capacity of pests and generate new ideas and a theoretical basis for environmentally friendly plant protection methods.

## Figures and Tables

**Table 1:** Changes in main energy substances between pre- and postmating in CK males (19 h after emergence) and DAT males (treated with 0.01% diallyl trisulfide for 7 h at 12 h after emergence).

	∆CK	∆DAT	*F*	*df*	*p*	Significance of Difference
Protein	66.9 ±5.62	49.18 ± 5.22	0.017	8	0.490	*
MAGPs	−1.41 ± 0.10	−0.35 ± 0.36	5.336	4	0.047	*
Triglyceride	−23.54 ± 6.55	3.77 ± 3.48	2.226	4	0.021	*
Glycogen	−7.11 ± 0.63	−8.94 ± 0.92	0.009	11	0.119	NS
Total soluble sugar	37.36 ± 2.08	42.68 ± 2.53	0.552	8	0.143	NS
Trehalose	−11.34 ± 0.44	−0.02 ± 0.28	1.497	14	<0.001	***
Trehalase	702.19 ± 69.71	−110.18 ± 117.89	1.954	14	<0.001	***

∆CK denotes the difference between “postmating” and “premating” values; ∆DAT denotes the difference between “postmating” and “premating” values. Data in the figure are presented as the mean ± SEM. NS, no significant difference, *, *p* < 0.05, ***, *p* < 0.001 (Tukey’s test).
